# Extending the Affinity Range of Weak Affinity Chromatography for the Identification of Weak Ligands Targeting Membrane Proteins

**DOI:** 10.3390/molecules28207113

**Published:** 2023-10-16

**Authors:** Adrien Deloche, François-Xavier Vidal, Lucile Jammas, Renaud Wagner, Vincent Dugas, Claire Demesmay

**Affiliations:** 1 Institut des Sciences Analytique, Universite Claude Bernard Lyon 1, ISA UMR 5280, CNRS, 5 Rue de la Doua, 69100 Villeurbanne, France; adrien.deloche@etu.univ-lyon1.fr (A.D.); francoisxaviervidal@live.fr (F.-X.V.); vincent.dugas@univ-lyon1.fr (V.D.); 2 Plateforme IMPReSs, CNRS UMR7242, Biotechnologie et Signalisation Cellulaire, Ecole Supérieure de Biotechnologie de Strasbourg, 67400 Illkirch, Francerenaud.wagner@unistra.fr (R.W.)

**Keywords:** membrane proteins, affinity chromatography, surface functionalization, weak-affinity interactions, adenosine receptor, fragment-based drug discovery

## Abstract

The identification of weak-affinity ligands targeting membrane proteins is of great interest in Fragment-Based Drug Design (FBDD). Recently, miniaturized weak affinity chromatography (WAC) has been proposed as a valuable tool to study interactions between small ligands and wild-type membrane proteins embedded in so-called nanodisc biomimetic membranes immobilized on GMA-co-EDMA monoliths in situ-synthesized in capillary columns (less than one microliter in volume). In this proof-of-concept study, the achievable affinity range was limited to medium affinity (low micromolar range). The present work investigates different strategies to extend the affinity range towards low affinities, either by increasing the density of membrane proteins on the chromatographic support or by reducing non-specific interactions with the monolith. The combination of the use of a new and more hydrophilic monolithic support (poly(DHPMA-co-MBA)) and a multilayer nanodisc grafting process (up to three layers) allows a significant increase in the membrane protein density by a more than three-fold factor (up to 5.4 pmol cm^−1^). Such an increase in protein density associated with reduced non-specific interactions makes it possible to extend the range of detectable affinity, as demonstrated by the identification and characterization of affinities of very low-affinity ligands (Kd values of several hundred micromolar) for the adenosine receptor AA_2A_R used as a model protein, which was not possible before. The affinity was confirmed by competition experiments.

## 1. Introduction

Identifying small molecules (called fragments) that interact with therapeutic proteins is the first (and, therefore, critical) step in Fragment-Based Drug Design (FBDD). This step consists of screening fragments against a purified protein using biophysical techniques, of which NMR, X-ray crystallography, and Surface Plasmon Resonance (SPR) are the most widely used [1,2,3,4,5,6,7,8]. In recent years, weak affinity chromatography has been proposed [9,10,11] as a viable alternative to these established methods, as it benefits from some interesting key features: (i) the protein target is immobilized, allowing it to be reused and reducing protein consumption; (ii) pools of up to several tens of fragments can be screened in a single experiment with mass spectrometric detection, reducing protein consumption and screening time [12]; and (iii) it is adapted to unpurified samples, which reduces the time and cost of sample preparation. However, fragment screening by weak affinity chromatography has been almost exclusively applied to soluble proteins [13,14,15].

Screening of membrane proteins (MPs) is more challenging, regardless of the method used. Two main strategies have been developed to make use of such highly sophisticated proteins for WAC applications, including (i) immobilization of cell membrane fragments, with the protein of interest surrounded by all other cell membrane components and (ii) use of purified unmodified protein stabilized in biomimetic membranes [16]. This latter strategy includes various approaches, such as the insertion of the MPs into immobilized artificial membranes (IAM), proteoliposomes (where the orientation of the protein through the membrane is random), or smaller supramolecular assemblies such as lipodisks or nanodiscs. In the latter two supramolecular assemblies, both the intra- and extra-cellular sides of the MPs embedded in nano-objects are exposed to the bulk solution so that the active site is always exposed to the surrounding media. It has been shown that lipodisks, which consist of a circular planar lipid bilayer stabilized by lipids modified with a polyethylene glycol head group that preferentially accumulate at the edge of the disk, can be a reliable membrane biomimetic environment for the study of integral MPs by WAC [17]. More recently, we have shown that miniaturized WAC (nano-WAC) can also be extended to highly valuable MPs embedded in biomimetic membranes such as nanodiscs [11]. The nanodisc is a supramolecular assembly containing the protein embedded in phospholipids (POPC and POPG) and surrounded by a biotinylated membrane scaffold protein (MSP). The miniaturization of the affinity column allows for a drastic reduction in the consumption of these supramolecular assemblies, which are difficult or very expensive to produce in large quantities but have proven to be valuable for the study of intact (wild-type) membrane proteins. We have thus demonstrated the proof of concept of using nano-WAC to screen fragments on MPs embedded in nanodiscs.

**Figure 1:**
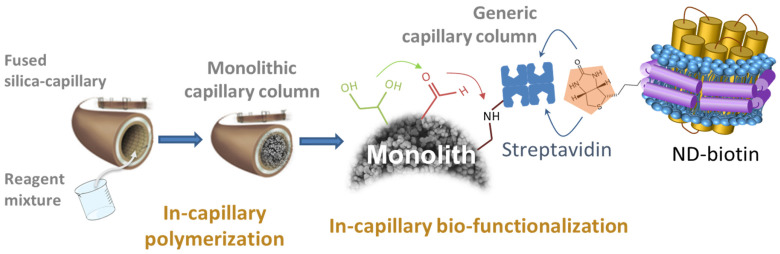
Schematic representation of the capillary column functionalized with nanodiscs (NDs).

The proposed methodology is based on the preparation of generic, universal, miniaturized, and in situ-synthesized monolithic columns modified with streptavidin (Figure 1). The (wild-type) target MP, stabilized in a biotinylated nanodisc as a biomimetic environment, is then immobilized on the generic column (in situ dynamic grafting) by the high-affinity streptavidin/biotin interaction. This grafting step is very rapid (which limits the degradation of fragile proteins) and reduces the amount of protein used to a strict minimum.

The proof of concept was carried out on a monolithic poly(GMA-co-EDMA) stationary phase using a model GPCR, Adenosine-A2A Receptor (AA_2A_R) [11]. With this monolith, the amount of so-called “active” or “binding” protein sites (*B_act_*) determined by performing affinity chromatography experiments with caffeine, a fragment of known affinity for the protein, was about 11.1 pmol (i.e., 1.3 ± 0.1 pmol cm^−1^). Regarding the density of binding sites (ratio of the number of binding sites (*B_act_*) and the volume of mobile phase in the column (*V_m_*)), we were able to detect fragments with medium affinity for AA_2A_R (caffeine, theophylline) but failed to detect other fragments (5-methoxy-1H-pyrrolo[3,2-b]pyridine (F468) and 5-methoxy-1H-pyrrolo[2,3-c]pyridine (F469)) identified by other screening methods [11]. Indeed, for such compounds, the non-specific retention due to hydrophobic effects with the monolithic support was too high when compared to the specific retention due to affinity (specific interaction). We estimated that the maximum detectable *K_d_* value was around 100 µM or even lower if non-specific interactions were not negligible. Such a K_d_ threshold is a limitation when very low affinities need to be detected, which is necessary in Fragment-Based Drug Design approaches. Indeed, some of the marketed drugs resulting from FBDD success stories were developed using very low-affinity starting “hit” fragments: fragments with *K_d_* of 200 µM for vemurafenib, 300 µM for venetoclax, and even up to 900 µM) [1]. Improving the performance of affinity columns is, therefore, highly desired to be able to detect fragments with greater K_d_ values, even in the presence of non-specific interactions that cannot be avoided.

In the present study, we first show by a brief rationale that a higher density of grafted proteins per unit volume combined to reduce non-specific interactions is necessary to extend the affinity range to higher *K_d_* values. We then present a detailed characterization of existing affinity columns based on poly(GMA-co-EDMA) monoliths generating non-specific hydrophobic interactions in order to propose a more adapted alternative setup to achieve our goal, i.e., the use of a more hydrophilic poly(DHPMA-co-MBA) monolith previously developed for hydrophilic interaction chromatography [18] combined with a multilayer nanodisc grafting strategy.

## 2. Nano-WAC Background and Rationale

The use and exploitation of weak affinity chromatography is based on the affinity of specific molecules for a protein immobilized on the support, measured by the association constant (K_a_) or dissociation constant (*K_d_*).

When working under conditions where the concentration of a given ligand in the mobile phase is well below its *K_d_* value for a protein, the retention factor (*k*), which corresponds to the ratio of the amounts of solute (i.e., ligand) in the stationary and mobile phases, is given by the following relationship (Equation (1)):

(1) $$k=\frac{1}{K_{d}}\times \frac{B_{act}}{V_{m}},$$

where *B_act_* is the number of active binding sites, and *V_m_* is the dead volume of the column.

Under these conditions and for a given column (given *B_act_*/*V_m_* ratio), a fragment that has no interaction with the protein will elute at the dead time (in zonal mode) or will break through at the dead time (in frontal mode). The greater the affinity of the fragment for the protein (i.e., the lower the *K_d_* of the ligand–protein complex), the greater the retention factor.

If the ligand concentration is of the same order of magnitude as the *K_d_* (0.1 *K_d_* < [*L*] < 10 *K_d_*), relationship 1 becomes:

(2) $$k_{\left[L\right]}=\frac{1}{K_{d}+[L]}\times \frac{B_{act}}{V_{m}},$$

where [*L*] is the ligand (fragment) concentration.

Under these conditions, the retention factor is no longer a constant and varies with the concentration of the ligand in the mobile phase. The retention time or breakthrough time will decrease with increasing ligand concentration.

In practice, the retention of fragments on an affinity column is generally not the result of specific interactions alone but, rather, a combination of specific interactions with the immobilized protein and non-specific interactions with the support. In the presence of non-specific interactions, relationship (2) becomes:

(3) $$k_{\left[L\right]}=\frac{1}{K_{d}+[L]}\times \frac{B_{act}}{V_{m}}+k_{nsi},$$

where *k_nsi_* is the retention factor for non-specific interactions (assuming that the ligand concentration is within the linear domain of the adsorption isotherm of the material).

To highlight the specific retention (affinity) of a compound for a given protein, its retention on a reference column (a column without protein or a column with a denatured protein) and on an affinity column can be compared. If no difference is observed, there is no affinity for the immobilized protein.

It has also been shown previously that the presence/absence of specific interactions can be highlighted by carrying out two experiments at two different and extreme ligand concentrations between 0.1 *K_d_* and 10 *K_d_* (e.g., ligand concentrations of 10 µM and 1000 µM for compounds with *K_d_* values ranging from a few tens of µM to a few hundreds of µM) [11]. If the compound does not interact specifically with the protein, no change in the retention factor (retention time, breakthrough time) will be observed. If the compound interacts specifically with the protein, a change in retention factor will be observed. For a given pair of concentrations, the higher the affinity (the lower the *K_d_*), the greater the variation in retention factor that is observed between the two concentrations. For a given pair of concentrations and a given *K_d_* value, the higher the protein active site density (*B_act_*/*V_m_*), the greater the variation in retention factor.

In fragment screening, the main concern is to be able to identify all fragments with specific affinities for the immobilized protein, and, in particular, those with very low affinities (i.e., high *K_d_* values), as discussed above. This is related to the ability to detect minute retention shifts on the chromatograms. If the limit of detection of the affinity is set at a variation of the retention factor of *x*% between the two concentrations under investigation, i.e., $k_{\left[L_{1}\right]}>\left(1+x\right)\times k_{\left[L_{2}\right]}$, the affinity detection threshold therefore depends on the *B_act_*/*V_m_* ratio, which must be maximized, and on the *k_nsi_*, which must be minimized.

Indeed, for a given set of screening parameters (affinity column and ligand concentrations), the non-specific retention factor affects the minimum affinity that can be detected. In order to detect the affinity, the following condition should be fulfilled:

(4) $$k_{nsi,limit}<\frac{\left(\frac{B_{act}/V_{m}}{K_{d}+\left[L_{1}\right]}-\left(1+x\right)\frac{B_{act}/V_{m}}{K_{d}+\left[L_{2}\right]}\right)}{x},$$

**Figure 2:**
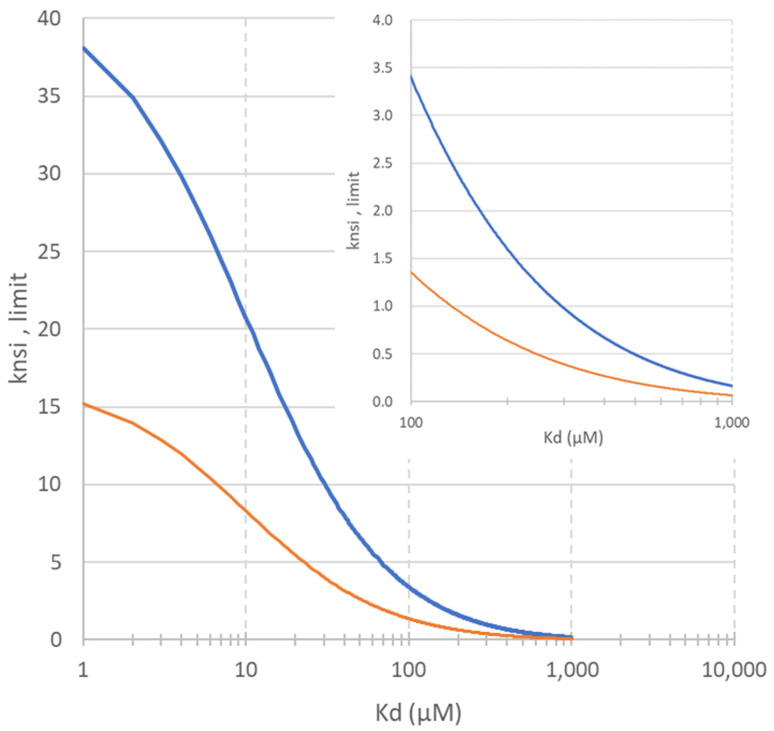
Graphic showing the variation of the maximum non-specific retention (*k_nsi_* limit) acceptable for a fragment of a given *K_d_* value (*K_d_* in µM). Experimental parameters used: x% set at 20%, 8.5 cm length column, and [*L*_1_] and [*L*_2_] were 10 and 1000 µM, respectively. Orange plot: *B_act_* = 12 pmol and blue plot: *B_act_* = 30 pmol. The insert is used to zoom in on the weak-affinity area.

For example, if two experiments are carried out at two extreme concentrations of 10 and 1000 µM, Figure 2 shows (*y*-axis) the maximum non-specific retention factor (*k_nsi, limit_*) that must not be exceeded to detect the affinity of a compound with a given *K_d_* (*x*-axis). This graph is given for two values of *B_act_*: 12 pmol for the 350 nL poly(GMA-co-EDMA) reference column (orange line) and a higher *B_act_* value of 30 pmol (blue line). Comparison of the two plots (orange and blue lines) clearly shows that an increase in *B_act_* (for a given column volume) allows lower affinities (higher *K_d_* values) to be detected, with fewer constraints on non-specific interactions.

Currently, using the *B_act_* value obtained with the poly(GMA-co-EDMA) monolith (12 pmol for a 350 nL column), only compounds with negligible non-specific interactions can be detected with low-affinity fragments (orange trace in Figure 2). For example, detection of a fragment with a *K_d_* of approximately 200 µM is achievable for fragments with a non-specific retention factor *k_nsi_* of less than 0. If we refer to a previous study on non-specific interactions with such a monolith, this limit cannot be reached (more than 50% of the fragments exceed this threshold for non-specific interactions [19]). At a higher *k_nsi_* value of 2, only fragments with a *K_d_* below 70 µM can be unambiguously detected.

These simulations show that it is essential to reduce non-specific interactions and increase the volume density of protein-binding active sites to make affinity chromatography a powerful tool for identifying low-affinity ligands of MPs inserted into nanodiscs. These objectives can be achieved either by modifying the monolithic support (for a more hydrophilic support with a larger specific surface area) or by modifying the nanodisc immobilization.

## 3. Results and Discussion

### 3.1. In-Depth Characterization of Poly(GMA-co-EDMA) Affinity Capillary Columns Grafted with NDs and Opportunities for Improvement

Poly(GMA-co-EDMA) monolithic affinity columns functionalized with NDs were prepared (see Figure 1) by direct capture of the biotinylated nanodiscs on the (generic) streptavidin-functionalized column. In order to choose the best strategy for increasing the density of active AA_2A_R binding sites (*B_act,AA2AR_*), the following questions need to be answered.

- Does the immobilization of NDs prevent access to the membrane protein embedded in NDs or lead to a loss of its binding properties?

This question can be answered by comparing the total number of NDs grafted (*B_tot,AA2AR_*) and the number of active binding sites (*B_act,AA2AR_*). The total number of NDs captured was measured by on-line spectrophotometric (UV-Vis) monitoring during ND percolation (breakthrough of NDs from the column is observed at 280 nm when the column is saturated with NDs). On poly(GMA-co-EDMA) monoliths, the total number of NDs captured (*B_tot,AA2AR_*) is 1.5 ± 0.2 pmol.cm^−1^ (Supplementary Material Figure S1). The amount of active AA_2A_R binding sites (*B_act,AA2AR_*) was determined in situ by frontal affinity chromatography using caffeine as a known test ligand (*K_d_* _caffeine/AA2AR_ = 23 µM) by plotting the inverse of the amount of caffeine captured (1/q_capt_) at different concentrations as a function of the inverse of the concentration of caffeine (1/[*L*]). The number of active AA_2A_R binding sites (*B_act,AA2AR_*) was equal to 1.3 ± 0.1 pmol.cm^−1^ (Supplementary Material Figure S2). These two values are not significantly different, indicating that immobilization does not alter the binding properties of proteins inserted into NDs.

- Is the number of streptavidin binding sites the limitation factor for capturing NDs?

Generic columns functionalized with streptavidin can be in situ-characterized by frontal affinity chromatography (nanoFAC-UV) to determine the number of streptavidin binding sites (*B_act,strepta_*) available using the same methodology as the one used for the determination of the number of AA_2A_R binding sites, except that 4′-Hydroxyazobenzene-2-carboxylic acid (HABA) is used as the known ligand of streptavidin (*K_d_*_HABA/streptavidin_ = 100 µM) (Supplementary Material Figure S3). On the poly(GMA-co-EDMA) monolith, the number of active binding sites capable of binding a biotin (*B_act,strepta_*) was measured to be 8.3 ± 0.3 pmol.cm^−1^ (*n* = 4 columns), which would theoretically allow an equivalent number of ND_S_ to be captured (provided that one ND binds one streptavidin binding site). On poly(GMA-co-EDMA) monoliths, the total number of NDs captured (*B_tot,AA2AR_*) was 1.5 ± 0.2 pmol cm^−1^, much lower than the number of streptavidin interaction sites present on the support (ratio 1/5, ND/streptavidin binding site). Given that each ND has an average of four to five biotins (MSP biotinylation rate = 2.3 and two MSPs per ND), the limitation in the number of captured nanodiscs could be due to the multivalency of the ND grafting (one ND occupying multiple streptavidin binding sites). To validate or invalidate this hypothesis, the number of residual streptavidin active binding sites (*B_act,strepta_*) was measured again after the ND grafting step. After the ND grafting step, 5.0 ± 0.7 pmol.cm^−1^ of streptavidin active binding sites remained on the support. This shows that 3 pmol cm^−1^ of streptavidin binding sites are occupied by 1.5 pmol cm^−1^ of ND, i.e., that each ND occupies an average of two active streptavidin sites (multivalency of two). This result also shows that there are still a large number of active streptavidin binding sites available on the support and that the limitation in the number of NDs captured is not due to a lack of active streptavidin binding sites but more likely to steric hindrance issues. This could be explained by the relatively large size of NDs compared to streptavidin. Indeed, the NDs used have a diameter of approximately 13 nm, whereas the size of streptavidin is approximately 5 nm in diameter [20].

- Is the spacer arm between the NDs and the biotin long enough to minimize steric hindrance?

Increasing the length of the spacer arm between the biotin and NDs could allow the reagent to form a long and flexible link, reducing the steric hindrance to binding to streptavidin molecules. Meanwhile, the spacer arm promotes ND rotation and the favorable orientation of the immobilized AA_2A_R binding site [21]. Spacer arm length refers to the distance between conjugated molecules and is usually classified as short (<10 Å), medium (10.1–30 Å), or long (>30 Å) [22]. NDs were prepared using membrane scaffold proteins (MSPs) biotinylated with two spacer arms of different sizes, 29 and 56 Angstroms for the short PEG4 arm and the long PEG12 arm, respectively. A slight (approximately 15%) and not significant (given the variation coefficient of about 20%) increase in *B_tot,A2AR_* and *B_act,A2AR_* was observed with a longer spacer arm (Supplementary Material Figure S4).

From the answers to these various questions, it appeared that only by changing the nature of the monolith and/or the ND grafting pathway would it be possible to increase the amount of NDs grafted.

### 3.2. Evaluation of a More Hydrophilic Monolith for ND Immobilization

To reduce non-specific interaction, we envisioned the use of poly(DHPMA-co-MBA), a more hydrophilic monolith, synthesized with a diol acrylate monomer (2,3-Dihydroxypropyl methacrylate, DHMPA) and a more hydrophilic crosslinker (Methylene bis acrylamide, MBA). This hydrophilic monolith offers reduced preparation time (the DHMPA is a diol monomer that eliminates the epoxy ring opening step, and its thermal polymerization at 87 °C takes only 30 min) and increased hydrophilicity, i.e., reduced non-specific interactions due to the hydrophobic effect [18]. The immobilization of NDs was performed using the same strategy as for the poly(GMA-co-EDMA) monolith. The number of active streptavidin binding sites, AA_2A_R binding sites, and grafted NDs were determined and compared to those obtained with the poly(GMA-co-EDMA) monolith. Results are summarized in Table 1.

Firstly, this new monolith fulfills the key performance criteria by allowing a significant increase in both the number of active streptavidin binding sites and the number of NDs (total or active). *B_act,strepta_* before grafting NDs, *B_tot,AA2AR_* and *B_act,AA2AR_* have been found to increase approximately twofold. The ratio between the number of active AA_2A_R binding sites and the total number of grafted NDs (*B_act,AA2AR_/B_tot,AA2AR_*), which represents the binding activity of NDs, remains high (90%) and unchanged when compared to that obtained with the previous monolith. With a binding activity of up to 90%, it can be assumed that each ND contains a protein capable of binding to its ligands. This result demonstrates that the poly(DHMPA-co-MBA) monolith is well suited for both the grafting of NDs and the biological binding properties of MPs. Again, it is worth noticing that the number of streptavidin active binding sites available after the grafting of NDs is still important. Up to 60% of the initial streptavidin binding sites remain free after the grafting of NDs on both monoliths. For the poly(DHMPA-co-MBA) monolith, 3.2 pmol cm^−1^ of NDs are grafted over the initial 14.6 pmol cm^−1^ streptavidin binding sites. Knowing that 8.7 pmol.cm^−1^ of active streptavidin binding sites remain on the support after the ND grafting, it can be deduced that 6 pmol cm^−1^ of streptavidin binding sites are occupied by 3.2 pmol cm^−1^ NDs, which are grafted in a divalent manner, i.e., each ND occupies an average of two active streptavidin sites (multivalency of two). The increase in the number of AA_2A_R binding sites on the poly(DHMPA-co-MBA) monolith is, thus, directly related to the increase in the number of active streptavidin binding sites. Again, steric hindrance is expected to limit the ratio of NDs to streptavidin binding sites, as it remains constant. This result shows that the poly(DHMPA-co-MBA) monolith has a higher specific surface area, which, as expected, allows a higher ND density to be achieved. To further increase the ND density, a multi-layer approach was then considered.

### 3.3. Multilayer Grafting

**Figure 3:**
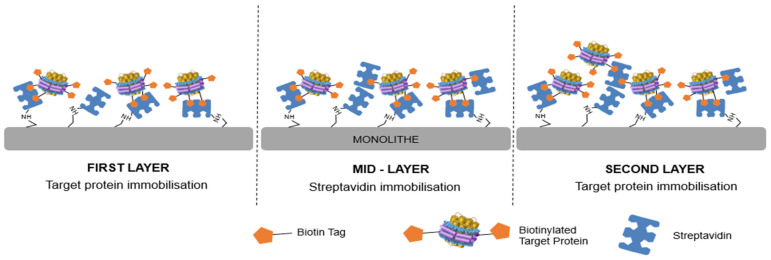
Schematic illustration of streptadivin–biotin multilayer immobilization process on a monolithic column.

In order to further increase the number of active AA_2A_R binding sites, a three-dimensional molecular assembly of the NDs has been considered. The relatively high biotin incorporation ratio (>5) on NDs is exploited in such an approach. Indeed, after a first grafting step of NDs on the streptavidin generic column (layer 1), some residual biotin is still present on the NDs (at least three biotins, as the grafting is bivalent, as shown in the previous section). These residual biotin residues are available to capture a new streptavidin layer. A solution of streptavidin was percolated, and the grafting was monitored at 280 nm. This second grafting step forms a mid-layer of streptavidin. As streptavidin has four active sites, it is expected that this streptavidin mid-layer is able to capture biotinylated NDs again (layer 2). This layering process can be repeated several times, as shown in Figure 3.

**Figure 4:**
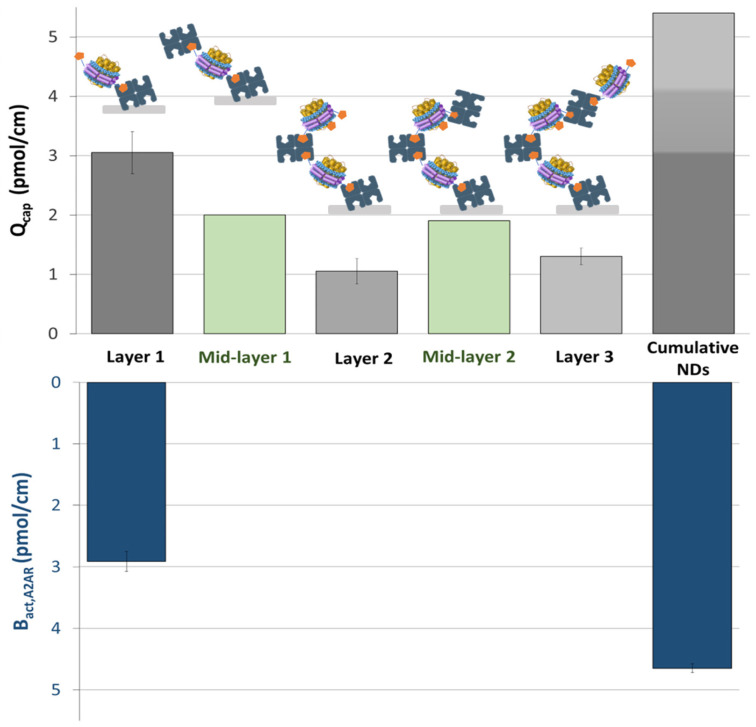
Plot representing the evolution of the quantity of AA2AR NDs and streptavidin (B_tot_, above) and the evolution of the quantity of AA2AR active sites (*B_act_*, AA2AR, below) added on 2 monolith columns at each step of a 3-step grafting process.

This multi-layer process was applied to the poly(DHMPA-co-MBA) monolith. For each ND layer or streptavidin intermediate layer, the total amount of streptavidin or captured NDs (pmol cm^−1^) was determined by monitoring the capture with UV spectrophotometric detection. The amount of active AA_2A_R binding sites was measured by nano-FAC experiments after the first and third layers. Results are summarized in Figure 4. Using the poly(DHPMA-co-MBA) monolith, the first step of ND grafting leads to a B_tot,AA2AR_ of approx. 3 pmol cm^−1^. Approximately 2 pmol cm^−1^ of streptavidin (i.e., 8 pmol cm^−1^ of biotin interaction sites) is captured by the free biotins of the NDs of the first layer. This confirms that free biotins are still present and available after the first ND layer. A further ND layer of 1.1 pmol cm^−1^ is captured on this streptavidin interface. The same behaviour was observed for the second mid-layer of streptavidin (addition of 1.9 pmol cm^−1^) and the third layer of NDs (1.3 ± 0.1 pmol cm^−1^). As already observed for the first layer, only a small fraction of the added biotin interaction sites are able to capture an ND due to steric hindrance. The cumulative amount of NDs after three layers is 5.4 pmol cm^−1^, i.e., 43 pmol for a column length of 8 cm. Meanwhile, the number of active AA_2A_R binding sites reaches 4.6 pmol cm^−1^ (vs. 2.9 for the first layer). This represents an approximately 60% increase compared to the first layer. It should also be noted that the proportion of active AA_2A_R binding sites is kept constant after the third layer (86.1 ± 1.3%), confirming that the NDs closest to the surface (layer 1) are still accessible to their ligands, even after the addition of several ND layers.

This increase in the number of MP binding sites (a gain of up to a factor of three over the poly(GMA-co-EDMA) monolith) and the reduction in non-specific interactions should allow the affinity range to be extended to lower affinities. With such amount of active binding sites and considering the simulation proposed in the rationale, the detection of low-affinity fragments should now be possible for compounds with higher non-specific interactions. For example, detection of a ligand with a *K_d_* as high as 250 µM should be achievable for ligands with a non-specific retention factor *k_nsi_* of 2. For compounds with negligible non-specific interactions, the affinity range should be extended to *K_d_* = 1 mM.

### 3.4. Identification of “Very” Weak-Affinity Fragments

In order to verify whether the poly(DHPMA-co-MBA) monolithic columns, with a higher density of active sites and reduced non-specific interactions, are able to identify weak-affinity fragments for AA_2A_R and have non-negligible non-specific interaction for the column, frontal mode affinity chromatography experiments were performed with two fragments described as potential AA_2A_R ligands [8]: 5-methoxy-1H-pyrrolo[3,2-b]pyridine (F468) and 5-methoxy-1H-pyrrolo[2,3-c]pyridine (F469). It should be noted that all the experiments carried out with these fragments on poly(GMA-co-EDMA) columns grafted with AA_2A_R NDs were unsuccessful due to excessive non-specific retention, which hindered the detection of affinity if affinity was present.

**Figure 5:**
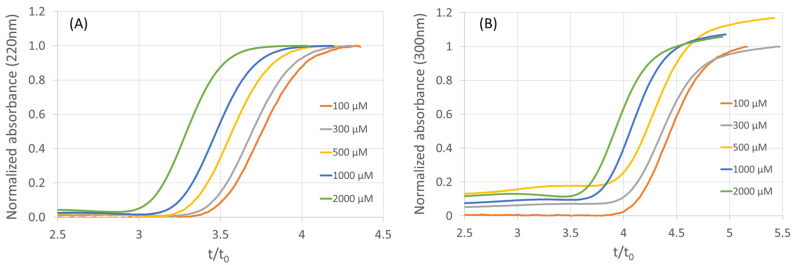
Frontal affinity chromatograms of 5-methoxy-1H-pyrrolo[3,2-b]pyridine (**A**) and 5-methoxy-1H-pyrrolo[2,3-c]pyridine (**B**) poly(DHPMA-co-MBA) monolith functionalized with 3 layers of AA_2A_R NDs. Column length 8.5 cm, mobile phase ammonium acetate 20 mM.

The two fragments were percolated at different concentrations (ranging from 5 µM to 2 mM) on a poly(DHPMA-co-MBA) monolith functionalized with AA_2A_R NDs (three layers). Breakthrough curves obtained are represented in Figure 5. For both compounds, the decrease in reduced breakthrough time (breakthrough time/dead time) with an increasing concentration is characteristic of affinity interactions. *K_d_* values (210 µM and 190 µM for 5-methoxy-1H-pyrrolo[3,2-b]pyridine and 5-methoxy-1H-pyrrolo[2,3-c]pyridine, respectively) were estimated according to Equation (3), with a *B_act_* value of 38 pmol/column and a dead volume of 350 nL. The *k_nsi_* values (2.28 and 2.88 for 5-methoxy-1H-pyrrolo[3,2-b]pyridine and 5-methoxy-1H-pyrrolo[2,3-c]pyridine, respectively) were estimated from the breakthrough times at high concentration (2 mM), where non-specific interactions largely predominate over specific ones.

This result shows that even in the presence of non-negligible non-specific interaction (*k_nsi_* higher than 2), we are able to detect very weak affinity and to give an estimation of the *K_d_* value.

**Figure 6:**
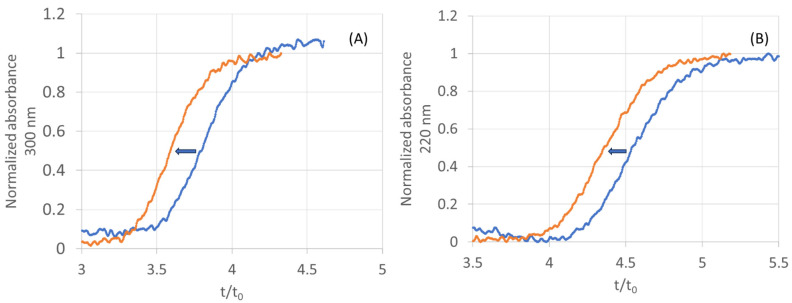
Frontal affinity chromatograms on poly(DHPMA-co-MBA) functionalized with AA_2A_R nanodiscs (3 layers, *B_act_* = 4.6 pmol cm^−1^). (**A**) Percolation of 5-methoxy-1H-pyrrolo[3,2-b]pyridine (F 468) at 10 µM in absence (blue plot) and presence (orange plot) of 50 µM of theophylline as competitor and (**B**) percolation of 5-methoxy-1H-pyrrolo[2,3-c]pyridine (F 469) at 10 µM in absence (blue plot) and presence (orange plot) of 50 µM of theophylline as competitor. Column length 8.5 cm.

Competition experiments were then performed with theophylline (theophylline, *K_d_* = 25 µM [23]) as the competitor (to confirm that the breakthrough time shift is due to affinity). The addition of a competitor at a concentration of 50 µM in the mobile phase resulted in a shift of the breakthrough time to a lower value for both compounds (Figure 6). Such a result confirms that these two fragments have affinity for AA_2A_R and that the interaction takes place at the same binding site as theophylline, i.e., in the orthosteric site.

## 4. Materials and Methods

### 4.1. Reagents and Buffers

Streptavidin (from Streptomyces avidinii, affinity purified, ≥13 U mg−1 of protein), (3-methacryloxypropyl)-trimethoxysilane (γ-MAPS), ethylene dimethacrylate (EDMA), N,N′-Methylenebis(acrylamide) (MBA), glycidyl methacrylate (GMA), acrylamide, 1-propanol, 1,4-butanediol, dimethyl sulfoxide (DMSO), sodium periodate, lithium hydroxide, dipotassium hydrogen phosphate (K_2_HPO_4_), o-phosphoric acid, sulfuric acid, sodium cyanoborohydride, triethylamine (TEA), azobis(isobutyronitrile) (AIBN), theophylline, caffeine, 4′-Hydroxyazobenzene-2-carboxylic acid (HABA), were purchased from Sigma-Aldrich (L’Isle d’Abeau Chesne, France). 2,3-Dihydroxypropyl methacrylate (DHPMA) was purchased from Polysciences (Hirschberg, Germany). 5-methoxy-1H-pyrrolo[3,2-b]pyridine (F468) and 5-methoxy-1H-pyrrolo[2,3-c]pyridine (F469) were a gift from I. Krimm. All aqueous solutions were prepared using >18 MΩ deionized water. Phosphate buffer was prepared by dissolving 1.17 g of K_2_HPO4 in 100 mL of ultrapure water, and the pH was adjusted to 7.4 with phosphoric acid.

### 4.2. Biochemistry Methods

#### 4.2.1. Production and Purification of AA_2A_R

The human AA_2A_R receptor N-terminally fused to the flag and decahistidine tags was produced and purified from a recombinant *Pichia pastoris* clone as previously described [11]. Briefly, whole-membrane preparations from yeast were diluted to approximately 2 mg.mL^−1^ in cold solubilization buffer (50 mM HEPES pH 7.4, 500 mM NaCl, 0.5% n-Dodecyl-ß-D-Maltopyranoside (β-DDM) (*w*/*v*), 0.05% Cholesteryl Hemisuccinate (CHS) (*w*/*v*), 30 mM imidazole, 1 µM 1,3-Dipropyl-8-cyclopentylxanthine (DPCPX), 0.3 mM EDTA, one antiprotease tablet) and incubated at room temperature for 30 min. After a centrifugation step at 100,000× *g* for 30 min, solubilized proteins were injected onto a 1 mL HisTrap HP column (Cytiva, Marlborough, MA, USA) at 1 mL.min^−1^ flow rate. The column was extensively washed with a 50 mM HEPES pH 7.4, 500 mM NaCl, 0.05% β-DDM (*w*/*v*), 0.005% CHS (*w*/*v*), 30 mM imidazole, 1 µM DPCPX buffer, and the proteins were eluted with a linear gradient from 30 to 500 mM imidazole. Fractions eluting at around 100 mM imidazole were pooled and injected onto an HiLoad Superdex 200 Increase 16/600 PG column (Cytiva, Marlborough, MA, USA). Proteins were separated at 1 mL.min^−1^ with a 50 mM HEPES pH 7.4, 150 mM NaCl, 0.02% β-DDM (*w*/*v*), 0.002% CHS (*w*/*v*) 1 µM DPCPX buffer. Fractions corresponding to the monomeric AA_2A_R receptor in detergent were pooled, concentrated to about 0.8 mg/mL on a Vivaspin20 30K MWCO device (Sartorius, Bangkok, Thailand), and directly used for reconstitution into lipid nanodiscs.

#### 4.2.2. Membrane Scaffold Protein Purification and Biotinylation

The membrane scaffold protein MSP1E3D1(-) was produced and purified from *E. coli* as previously described [11]. The MSP1E3D1(-) was then in vitro biotinylated via an incubation with either of the Thermo Scientific reagents EZ-Link^TM^ NHS-PEG4-Biotin (short spacer arm) or EZ-Link^TM^ NHS-PEG12-Biotin (long spacer arm) at a molar ratio of 1:5 in a 20 mM HEPES pH 7.4, 100 mM NaCl, 0.5 mM EDTA buffer. The reaction was stopped by the addition of 5 mM Tris-HCl pH 7.4 after 1 h incubation at room temperature. The biotinylated-MSP1E3D1(-) was then separated from the free reagent in a final desalting step (HiTrap desalting, Cytiva, Marlborough, MA, USA) in a 20 mM Tris-HCl pH 7.4, 100 mM NaCl, 0.5 mM EDTA storage buffer, snap-frozen in liquid nitrogen, and stored at −80 °C until use. The biotinylation was confirmed in a standard Western blot revealed with extravidin-HRP, and the mean biotinylation ratio was determined by MALDI-TOF-MS.

#### 4.2.3. Nanodisc Assembly and Purification

The biotinylated MSP1E3D1(-) was mixed at a 1:70 molar ratio with purified lipids (POPC/POPG; 3/2 molar ratio) previously dissolved at 24 mM in a 50 mM HEPES pH 7.4, 150 mM NaCl, 48 mM Na-cholate buffer. The MSP:lipid mixture was incubated for 15 min on ice before the purified receptor was added at a molar ratio of 1:10 receptor:MSP1E3(-) and incubated for a further 60 min on ice. Self-assembly was initiated by detergent removal using BioBeads SM-2 (Biorad, Hercules, CA, USA) added at 0.25 g per mL of reconstitution mixture. After an incubation overnight at 4 °C on a tube rotator, the Biobeads were removed by centrifugation, and the recovered supernatant was directly injected on a 1 mL HisTrap HP column (Cytiva, Marlborough, MA, USA) previously equilibrated in a 50 mM HEPES pH 7.4, 300 mM NaCl, 10 mM imidazole buffer. After extensive washing with the equilibration buffer, the AA_2A_R-containing discs were eluted with the same buffer in the presence of 500 mM imidazole. The eluted fractions were then pooled and applied to a Superdex 200 Increase 10/300 GL column (Cytiva, Marlborough, MA, USA) previously equilibrated in a 50 mM HEPES pH7.4, 150 mM NaCl buffer. Elution was performed at 0.3 mL.min^−1^, and the fractions containing the pure AA_2A_R nanodiscs were finally aliquoted, snap-frozen in liquid nitrogen, and stored at −80 °C (full characterization of the NDs (TEM image, activity…) is provided in [11]).

### 4.3. Monolithic Capillary Column Synthesis

75 µm i.d. fused-silica capillaries with polyimide (TSP) or with UV-transparent coating (TSH) coating were purchased from Cluzeau info Labo (Sainte-Foy-La-Grande, France). Silica activation was performed by flushing 15 cm long capillaries with a 5% (*v*/*v*) solution of γ-MAPS in methanol/water (95/5, *v*/*v*) and 2.5% TEA for 1 h at 7 bars. The capillaries were then rinsed with methanol for 15 min at 7 bar and dried at room temperature under a nitrogen stream prior to use.

#### 4.3.1. In-Capillary Poly(GMA-co-EDMA) Monolith Synthesis

Poly(GMA-co-EDMA) monoliths were synthesized as described in a previous work. The polymerization mixture was prepared by mixing 0.9 mL GMA, 0.3 mL EDMA, 1.05 mL 1-propanol, 0.6 mL 1,4-butanediol, 0.15 mL ultrapure water, and 12 mg of AIBN initiator. The pre-treated TSH capillary was filled with the polymerization mixture under 1 bar N_2_ pressure. The photopolymerization was performed in a Bio-link UV crosslinker (VWR International, Rosny-sous-Bois, France) under 365 nm UV light with a total energy of 6 J cm^−1^. A PEEK tube (380 μm i.d.) was used as a mask to cover non-irradiated areas. After polymerization, the capillary was rinsed with methanol for 1 h.

#### 4.3.2. In-Capillary Poly(DHPMA-co-MBA) Monolith Synthesis

Poly(DHPMA-co-MBA) monoliths were prepared as follows. First, a solvent mixture was prepared by mixing 888 mg DMSO, 395 mg 1,4-butanediol, and 397 mg dodecanol. Then, 80 mg of MBA was added and sonicated for 1 h at room temperature. After the addition of 120 mg of DHPMA, the mixture was sonicated for 1 h at room temperature. Then, 2 mg of AIBN was added, and the final mixture was sonicated for 15 min at room temperature. The pre-activated fused silica capillary was filled with the polymerization mixture and sealed prior to polymerization in a water bath at 85 °C for 2 h. After polymerization, the monoliths were rinsed with methanol for 1 h and kept wet in water until use.

### 4.4. Column Biofunctionalization

#### 4.4.1. Preparation of Streptavidin-Functionalized Monolithic Generic Columns

Epoxy groups of GMA-based monolith were first hydrolyzed into diols in an acidic medium (by flowing 1 M sulfuric acid for 2 h). Diol monolithic columns (hydrolyzed GMA-based and DHPMA-based monoliths) were oxidized to aldehyde columns using a 0.12 M NaIO_4_ solution at pH 5.5 for 1 h at 7 bar. A 1 mg mL^−1^ streptavidin solution containing 4 mg mL^−1^ NaBH_3_CN solution in 67 mM phosphate buffer (pH 6) was then percolated through the column for 18 h at 7 bar and room temperature. After immobilization, the column was rinsed with sodium borohydride (2.5 mg mL^−1^, phosphate buffer 67 mM pH 8) (2 h, 7 bar) to reduce residual aldehydes. The streptavidin columns were then rinsed with phosphate buffer and stored at 4 °C.

#### 4.4.2. Immobilization of AA_2A_R Nanodiscs on Monolithic Capillary Columns

Immobilization was performed dynamically by simply flowing a few microliters of a dilute solution of nanodisc-embedded AA_2A_R (µM range) through the column. The immobilization step was monitored using in situ UV detection at 280 nm to stop the sample flow when saturation was reached (about a few tens of minutes, depending on the AA_2A_R concentration) and to quantify the total amount of nanodiscs captured (B_tot,AA2AR_).

#### 4.4.3. Multi-Layer Process

Columns with a first layer of NDs were dynamically grafted with streptavidin by flowing a solution of streptavidin at a concentration ranging between 5 and 15 μM in phosphate buffer, 67 mM, pH 7.4). The streptavidin capture was monitored by in situ UV detection at 280 nm (mid-layer). The process was then repeated with the same sample of biotinylated NDs as that used for the first-layer grafting. This layering process was repeated for up to 3 layers of NDs.

### 4.5. Nano-FAC Experiments

#### 4.5.1. Instrumentation

Frontal weak affinity chromatography experiments were performed on an Agilent 7100 capillary electrophoresis system (Agilent Technologies, Waldbronn, Germany) equipped with a diode array detector and an external nitrogen pressurization device to achieve pressures up to 12 bar. System control and data acquisition were performed using 3D-CE Chemstation software B.040.3-SP1 (Agilent, Santa Clara, CA, USA). All experiments were performed at 25 °C in the short-end injection mode, with the inlet of the capillary immersed in the solution to be percolated.

#### 4.5.2. Quantification of the Number of Protein Active Binding Sites B_act_ (Number of Streptavidin Binding Sites *B_act,strepta_* or Number of AA_2A_R Binding Sites *B_act,AA2AR_*)

4′-Hydroxyazobenzene-2-carboxylic acid (HABA) and caffeine (*K_d_* = 23 µM) were used in frontal affinity chromatography as known ligands of streptavidin (*K_d_* = 100 μM) and AA_2A_R, respectively. Frontal affinity experiments were performed in the staircase mode (i.e., without rinsing between percolations of different concentrations) by successively percolating 5, 10, 50, 100, and 200 μM HABA solutions or X, Y, and A µM caffeine solutions prepared in phosphate buffer, 67 mM, pH = 7. The double reciprocal plot of the amount of ligand captured versus the ligand concentration allows the determination of the number of available active binding sites (*B_act,strepta_* and *B_act,AA2AR_*).

## 5. Conclusions

In the present miniaturized weak affinity chromatography study, we demonstrate that the use of a new and hydrophilic monolithic support combined with a multilayer nanodisc grafting process (up to three layers) promotes a significant increase in the membrane protein density by a more than three-fold factor (up to 5.4 pmol cm^−1^). Such an increase in protein density associated with reduced non-specific interactions makes it possible to extend the range of detectable affinity, as demonstrated by the identification and characterization of affinities of very low-affinity ligands (*K_d_* values of several hundred micromolar) for the adenosine receptor, which was not possible before. Competition experiments confirm that the interaction of these ligands occurs in the orthosteric binding site of the adenosine receptor.

## Figures and Tables

**Table 1:** Comparison of the number of binding sites of AA_2A_R on poly(GMA-co-EDMA) and poly(DHPMA-co-MBA) monolithic columns at various stages of elaboration.

	Poly(GMA-co-EDMA)	Poly(DHPMA-co-MBA)
B_act,strepta_ (starting column)	8.2 ± 0.3 pmol cm^−1^	14.3 ± 1.7 pmol cm^−1^
B_tot,AA2AR_ (grafted NDs)	1.5 ± 0.2 pmol cm^−1^	3.1 ± 0.4 pmol cm^−1^
B_act,AA2AR_/binding activity	1.3 ± 0.1 pmol cm^−1^/~90%	2.9 ± 0.2 pmol cm^−1^/~90%
B_act,strepta_ (after grafting NDs)	5.0 ± 0.3 pmol cm^−1^	7.7 ± 0.4 pmol cm^−1^

## Data Availability

Data sharing is not applicable.

## Supplementary Materials

The following supporting information can be downloaded at: https://www.mdpi.com/article/10.3390/molecules28207113/s1. Figures S1 and S2 illustrate the graphical plots that allow the determination of the number of NDs grafted and the number of active AA_2A_R binding sites, respectively, by nano-frontal affinity chromatography. Figure S3 presents affinity chromatograms using HABA as a ligand to determine the number of active streptavidin binding sites. Figure S4 presents the influence of the spacer arm (PEG4 or PEG12) on the amount of active binding sites.

## References

[1] Erlanson D.A., Fesik S.W., Hubbard R.E., Jahnke W., Jhoti H. (2016). Twenty Years on: The Impact of Fragments on Drug Discovery. Nat. Rev. Drug Discov..

[2] Renaud J.-P., Chung C., Danielson U.H., Egner U., Hennig M., Hubbard R.E., Nar H. (2016). Biophysics in Drug Discovery: Impact, Challenges and Opportunities. Nat. Rev. Drug Discov..

[3] Chen D., Errey J.C., Heitman L.H., Marshall F.H., IJzerman A.P., Siegal G. (2012). Fragment Screening of GPCRs Using Biophysical Methods: Identification of Ligands of the Adenosine A_2A_ Receptor with Novel Biological Activity. ACS Chem. Biol..

[4] Congreve M., Rich R.L., Myszka D.G., Figaroa F., Siegal G., Marshall F.H. (2011). Fragment Screening of Stabilized G-Protein-Coupled Receptors Using Biophysical Methods. Methods in Enzymology.

[5] Aristotelous T., Ahn S., Shukla A.K., Gawron S., Sassano M.F., Kahsai A.W., Wingler L.M., Zhu X., Tripathi-Shukla P., Huang X.-P. (2013). Discovery of Β2 Adrenergic Receptor Ligands Using Biosensor Fragment Screening of Tagged Wild-Type Receptor. ACS Med. Chem. Lett..

[6] Shepherd C.A., Hopkins A.L., Navratilova I. (2014). Fragment Screening by SPR and Advanced Application to GPCRs. Prog. Biophys. Mol. Biol..

[7] Navratilova I., Hopkins A.L. (2010). Fragment Screening by Surface Plasmon Resonance. ACS Med. Chem. Lett..

[8] Igonet S., Raingeval C., Cecon E., Pučić-Baković M., Lauc G., Cala O., Baranowski M., Perez J., Jockers R., Krimm I. (2018). Enabling STD-NMR Fragment Screening Using Stabilized Native GPCR: A Case Study of Adenosine Receptor. Sci. Rep..

[9] Duong-Thi M.-D., Meiby E., Bergström M., Fex T., Isaksson R., Ohlson S. (2011). Weak Affinity Chromatography as a New Approach for Fragment Screening in Drug Discovery. Anal. Biochem..

[10] Tsopelas F., Tsantili-Kakoulidou A. (2019). Advances with Weak Affinity Chromatography for Fragment Screening. Expert Opin. Drug Discov..

[11] Lecas L., Hartmann L., Caro L., Mohamed-Bouteben S., Raingeval C., Krimm I., Wagner R., Dugas V., Demesmay C. (2020). Miniaturized Weak Affinity Chromatography for Ligand Identification of Nanodiscs-Embedded G-Protein Coupled Receptors. Anal. Chim. Acta.

[12] Calleri E., Temporini C., Caccialanza G., Massolini G. (2009). Target-Based Drug Discovery: The Emerging Success of Frontal Affinity Chromatography Coupled to Mass Spectrometry. ChemMedChem.

[13] Ohlson S., Duong-Thi M.-D. (2018). Fragment Screening for Drug Leads by Weak Affinity Chromatography (WAC-MS). Methods.

[14] Meiby E., Simmonite H., le Strat L., Davis B., Matassova N., Moore J.D., Mrosek M., Murray J., Hubbard R.E., Ohlson S. (2013). Fragment Screening by Weak Affinity Chromatography: Comparison with Established Techniques for Screening against HSP90. Anal. Chem..

[15] Duong-Thi M.-D., Bergström M., Fex T., Isaksson R., Ohlson S. (2013). High-Throughput Fragment Screening by Affinity LC-MS. J. Biomol. Screen..

[16] Ma W., Wang C., Liu R., Wang N., Lv Y., Dai B., He L. (2021). Advances in cell membrane chromatography. J. Chromatogr A.

[17] Duong-Thi M.-D., Bergstrom M., Edwasrds K., Eriksson J., Ohlson S., To Yiu Ying J., Torres J., Agmo Hernandez V. (2016). Lipodisks Integrated with Weak Affinity Chromatography Enable Fragment Screening of Integral Membrane Proteins. Analyst.

[18] Gil J., Passalacqua G., Deloche A., Vidal F.-X., Dugas V., Demesmay C. (2023). Optimization of the Preparation of Hydrophilic Poly(DHPMA-Co-MBA) Monolithic Capillary Columns: A New Support for Affinity Chromatography. Separations.

[19] Gil J., Krimm I., Dugas V., Demesmay C. (2023). Preparation of Miniaturized Hydrophilic Affinity Monoliths: Towards a Reduction of Non-Specific Interactions and an Increased Target Protein Density. J. Chromatogr. A.

[20] Kuzuya A., Numajiri K., Kimura M., Komiyama M. (2008). Single-Molecule Accommodation of Streptavidin in Nanometer-Scale Wells Formed in DNA Nanostructures. Nucleic Acids Symp. Ser..

[21] Chen J., Yu B., Cong H., Shen Y. (2023). Recent Development and Application of Membrane Chromatography. Anal. Bioanal. Chem..

[22] Shao J., Drioli E., Giorno L. (2015). Spacer Arm Length. Encyclopedia of Membranes.

[23] De Lera Ruiz M., Lim Y.-H., Zheng J. (2014). Adenosine A_2A_ Receptor as a Drug Discovery Target. J. Med. Chem..

