# Supplementary material for: Extending the Affinity Range of Weak Affinity Chromatography for the Identification of Weak Ligands Targeting Membrane Proteins
Source: Molecules. 2023 Oct 16;28(20):7113. doi: 10.3390/molecules28207113 (PMC10608817; doi:10.3390/molecules28207113)
Supplement: Supplementary file 1 [file molecules-28-07113-s001.zip › molecules-2624305-supplementary.pdf]

Extending the affinity range of Weak Affinity Chromatography for the identification of weak ligands targeting membrane proteins

Adrien Deloche [1], François-Xavier Vidal [1], Lucile Jammas[2], Renaud Wagner [2], Vincent Dugas [1] and Claire Demesmay [1,*]

[1] Université Claude Bernard Lyon 1, Institut des Sciences Analytiques, ISA UMR 5280, CNRS, 5 rue de la Doua, 69100 Villeurbanne, France 1; adrien.deloche@etu.univ-lyon1.fr (A.D.);
francoisxaviervidal@live.fr (F.-X.V.); vincent.dugas@univ-lyon1.fr (V.D.)

[2] Plateforme IMPReSs, CNRS UMR7242, Biotechnologie et Signalisation Cellulaire, Ecole Supérieure de Biotechnologie de Strasbourg, Illkirch, France; renaud.wagner@unistra.fr

* Correspondence: demesmay@univ-lyon1.fr.

Supplementary Material S1 :

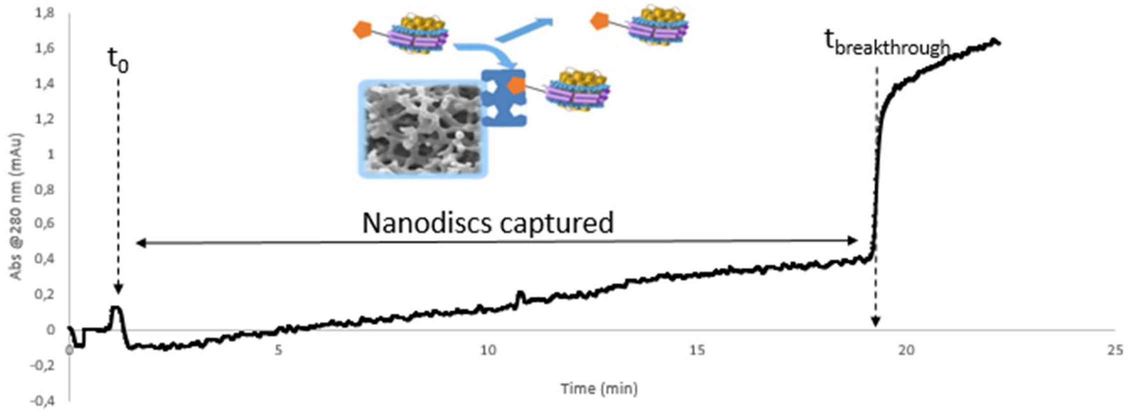

Figure S1. UV monitoring (@280 nm) of the capture of biotinylated $AA_{2A}R$ nanodiscs by the streptavidine monolith. $AA_{2A}R$ nanodiscs (2.6 µM) solubilized in 50 mM HEPES pH 7.4, 150 mM NaCl; streptavidin monolith l = 8.5 cm i.d 75 µM ; applied pressure 12 MPa (0.14 $cm.s^{-1}$); $t_{capture}=t_{breakthrough}-t_0$=18,3 min; amount of nanodiscs captured = 1.7 pmol/cm.

Supplementary Material S2 :

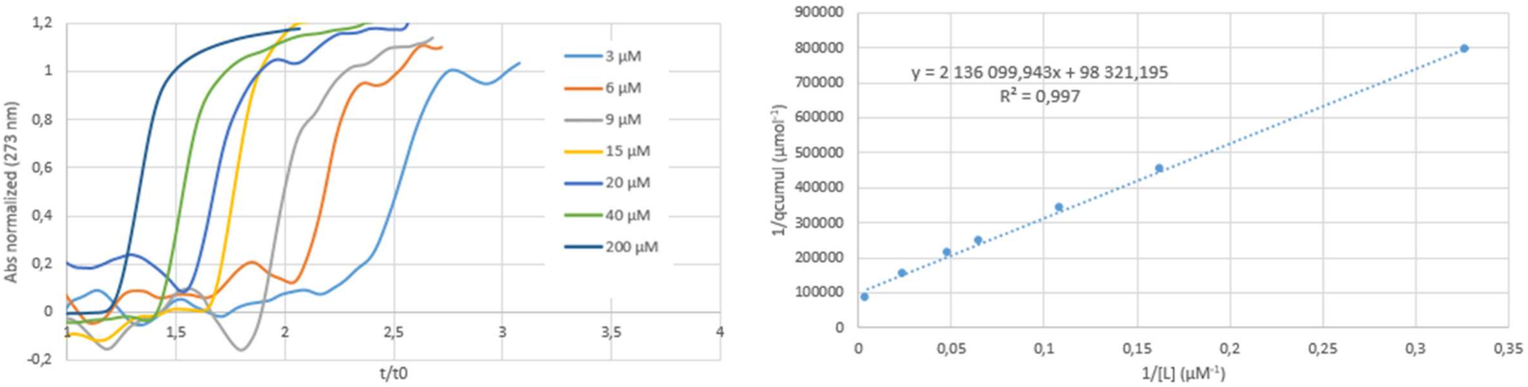

Figure S2. Frontal affinity chromatography experiments implemented to study the interaction between caffeine and immobilized $AA_{A2}R$- Caffeine solutions (solubilized in acetate buffer 20mM pH=7.4) are percolated at increasing concentrations ranging from 3 to 200µM on a poly(GMA-co- EDMA) monolith column functionalized with NDs; . Column dimension : l=8.5 cm, i.d = 75 µM; applied pressure 12 MPa (0.14 $cm.s^{-1}$).

Supplementary Material S3 :

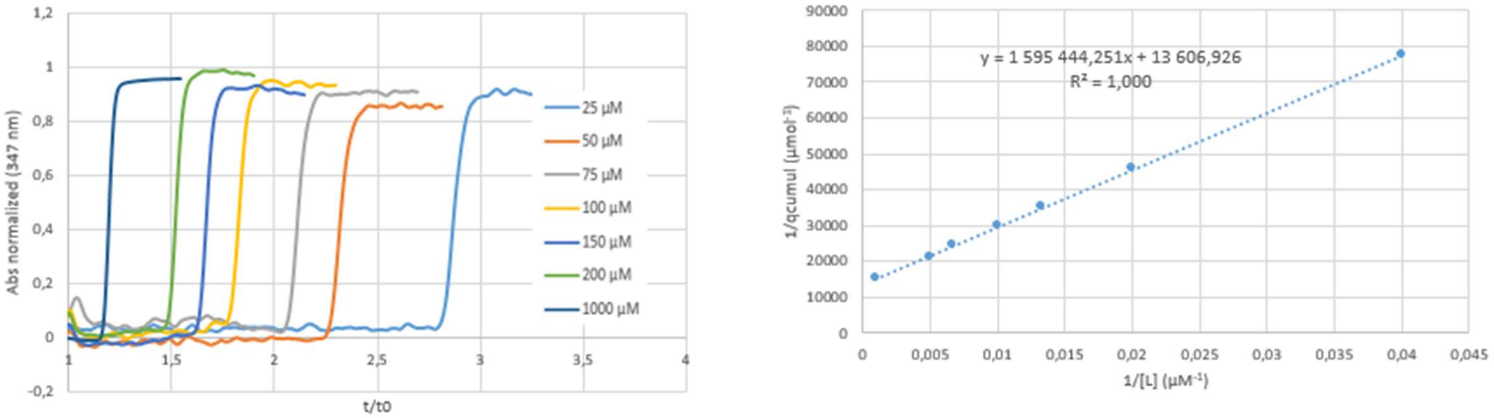

Figure S3. Frontal affinity chromatography analysis of the interaction between HABA and immobilized streptavidin on poly(GMA-co- EDMA) monolith column. Column dimension : l=8.5 cms i.d = 75 µM; applied pressure 12 MPa (0.14 $cm.s^{-1}$).

Supplementary Material S4 :

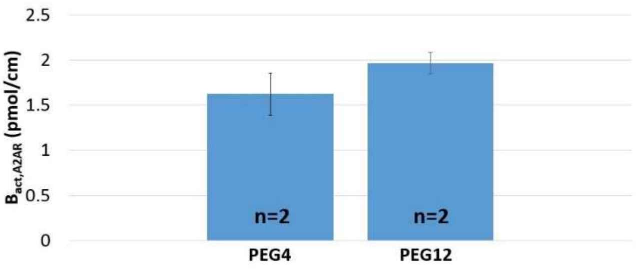

Figure S4. Comparison of the number of active binding sites determined on monolithic capillary columns using $AA_{A2}R$ nanodiscs functionalized with biotin-PEG4 and biotin-PEG 12 linkers.
